# *OsNUDX23* regulates early seed germination by modulating ROS balance and starch metabolism in rice

**DOI:** 10.3389/fpls.2025.1581800

**Published:** 2025-06-13

**Authors:** Xing Dai, Wenwen Kong, Feiyan Wu, Zijie Pan, Lei Gao, Beixin Mo, Yu Yu, Weigui Luo

**Affiliations:** ^1^ Guangdong Provincial Key Laboratory for Plant Epigenetics, Longhua Bioindustry and Innovation Research Institute, College of Life Sciences and Oceanography, Shenzhen University, Guangdong, Shenzhen, China; ^2^ Key Laboratory of Optoelectronic Devices and Systems of Ministry of Education and Guangdong Province, College of Optoelectronic Engineering, Shenzhen University, Shenzhen, China; ^3^ Beijing Advanced Center of RNA Biology (BEACON), State Key Laboratory for Protein and Plant Gene Research, Peking-Tsinghua Joint Center for Life Sciences, School of Life Sciences, Peking University, Beijing, China; ^4^ Lushan Botanical Garden, Chinese Academy of Sciences, Jiujiang, China

**Keywords:** *OsNUDX23*, seed germination, Nudix hydrolase, ROS homeostasis, starch metabolism

## Abstract

Seed germination is a complex biological process that encompasses the mobilization of stored nutrients, the resumption of metabolic activities, and the responses to various environmental stimuli. Reactive Oxygen Species (ROS) play a dual role during seed germination: at low concentrations, they function as signaling molecules to facilitate germination, while at high levels, they can induce oxidative damages. Therefore, maintaining ROS homeostasis through scavenging mechanisms is crucial for optimal seed germination. In this study, we investigated the role of *OsNUDX23* in rice seed germination. Our findings indicated that OsNUDX23 acts as an active Nudix hydrolase towards diverse substrates including NAD, NADH, NADPH, FAD, and ADPG. Loss-of-function *OsNUDX23* mutants displayed earlier coleoptile elongation, delayed radicle elongation and reduced germination rate during post-imbibition stages when compared to wildtype plants, suggesting its intricate role during seed germination. Transcriptome analyses revealed that *OsNUDX23* influences the expression of genes involved in ROS metabolism and starch metabolism pathways. Further investigation revealed that OsNUDX23 inhibits the activity of NADPH oxidase (NOX) and reduces the accumulation of NADP^+^, resulting in elevated ROS levels in *Osnudx23* mutants. Down-regulation of genes involved in starch and sucrose metabolism was observed in *Osnudx23* mutants during post-imbibition, accompanied with accumulated starch content. Collectively, these results demonstrate that *OsNUDX23* plays a critical role in rice seed germination by scavenging ROS to maintain redox balance and modulating starch metabolism.

## Introduction

Seed germination, responsible for the transformation from seed to seedling, is a key initiation step in plant growth (Shu et al., 2015). This process initiates with seed imbibition, which triggers enzymatic activation and engages metabolic pathways, ultimately leading to radicle emergence (Wang et al., 2020). The complex germination process is regulated by an intricate network of molecular mechanisms, encompassing transcriptional regulation, hormonal modulation, metabolic pathway activation, and antioxidant system induction (Farooq et al., 2022).

Reactive Oxygen Species (ROS) play a pivotal role in seed germination by modulating dormancy release, initiating germination, and mediating responses to environmental stimuli (Gomes and Garcia, 2013; Wang et al., 2024b). As essential components of cellular redox networks, ROS — including superoxide anion (O_2_^-^), hydrogen peroxide (H_2_O_2_) and hydroxyl radicals (·OH) — are present throughout the seed life cycle (Leymarie et al., 2012; Moon et al., 2003; Rouhier, 2010). NADPH oxidases (NOXs), which are localized on the plasma membrane, constitute an important source of ROS generation in plants (Sagi and Fluhr, 2006; Suzuki et al., 2011). NOXs catalyze the production of O_2_^-^ by transferring an electron from intracellular NADPH across the membrane to molecular oxygen (O_2_) via flavin adenine dinucleotide (FAD). ROS serve a dual function, they act as signaling molecules to regulate respiratory metabolism, nutrient mobilization, and other physiological processes; at higher concentrations, ROS can directly cleave cell wall polysaccharides, thereby loosening the plant cell wall and facilitating germination (Dietz et al., 2010; Mittler et al., 2011). Excess levels of ROS can induce oxidative damage to lipids, proteins, and nucleic acids, which may disrupt essential cellular functions and the hormonal balance required for germination (Bailly, 2004; Farooq et al., 2021). Therefore, maintaining an optimal balance of ROS is essential for successful seed germination. This balance is achieved by activation of both enzymatic and non-enzymatic antioxidant defense systems (Bailly, 2004). Enzymatic antioxidants such as superoxide dismutase (SOD), peroxidase (POD), catalase (CAT), and ascorbate peroxidase (APX) play pivotal roles in the plant defense system. Non-enzymatic antioxidants, including carotenoids, α-tocopherol, glutathione (GSH) and ascorbate (ASA), further contribute to this defense. For instance, carotenoids accumulate to against ROS and promote antioxidant activity during seed germination (Nguyen et al., 2023; Wang et al., 2024a). Together, these complementary systems maintain cellular redox homeostasis and protect cells from oxidative stress, playing a critical role in ensuring successful seed germination.

Starch metabolism plays a vital role in seed germination by supplying essential energy and carbon skeletons required for embryo development (Damaris et al., 2019; Sugimoto et al., 1998). Enzymes such as amylases and glucosidases catalyze the degradation of starch into simpler sugars, which are subsequently metabolized to provide energy (Damaris et al., 2019; Karrer and Rodriguez, 1992; Muñoz-Llandes et al., 2023). In rice, there are eight functional α-amylases: *Amy1A*, *Amy1C*, *Amy2A*, *Amy3A*, *Amy3B*, *Amy3C*, *Amy3D*, and *Amy3E* (Mitsui et al., 1996; Nanjo et al., 2004). *RAmy1A-B* and *RAmy3D* are expressed in both the embryo and aleurone layer, whereas *Amy3B-C* and *RAmy3E* exhibit preferential expression in the aleurone layer (Karrer et al., 1991). Beta-glucosidases (EC 3.2.1.21, BGlus) hydrolyze the glycosidic bonds to release glucose and aglycones (Akiyama et al., 1998). Studies have showed that β-glucosidase activity increases during germination in rice. For example, *BGlu1* and *BGlu2* were highly expressed in the shoot during germination (Opassiri et al., 2003). The loss-of-function mutant *bglu10*, *bglu 24*, and *bglu 33* exhibited reduced germination rates (Ren et al., 2019).

The NUDIX (nucleoside diphosphate linked to another moiety, X) superfamily encompasses a large number of functionally diverse proteins, also referred as NUDT or NUDX (McLennan, 2006; Srouji et al., 2017). Within this superfamily, the largest group is the Nudix hydrolases, which exhibit distinct substrate specificities (Srouji et al., 2017). NUDX proteins are characterized by a conserved Nudix domain that facilitates the hydrolysis of various nucleoside diphosphate derivatives, including nucleoside di- and triphosphates, nucleotide coenzymes, nucleotide sugars, and RNA caps (Bessman et al., 1996; McLennan, 2006; Yoshimura and Shigeoka, 2015). Increasing evidences suggest that NUDX proteins play extended roles in physiological and biochemical processes such as metabolic homeostasis and development, some of which rely on their hydrolase activities (Liu et al., 2022; Ogawa and Yoshimura, 2018; Yoshimura and Shigeoka, 2015). In Arabidopsis, AtNUDX2 maintains NAD^+^ and ATP levels through the hydrolysis of ADP-ribose, thereby preventing excessive NAD^+^ and ATP depletion caused by poly (ADP-ribose) polymerase (PARP) activating poly(ADP-ribosyl)ation under oxidative stress. Overexpression of AtNUDX2 ultimately enhances cellular stress tolerance and reduces cell death (Ogawa et al., 2009). AtNUDT7 plays an important role in maintaining redox balance through the regulation of NADH pyrophosphohydrolase activity. Loss-of-function mutations in *AtNUDT7* lead to elevated levels of ROS and ABA, both of which inhibit seed germination (Zeng et al., 2014). In the absence of chloroplast-localized *AtNUDX19*, plants exhibit enhanced NADP-dehydrogenase activity in roots under both normal and arsenic-induced stress conditions, suggesting that AtNUDX19 plays a role in regulating NADPH levels and redox homeostasis (Corpas et al., 2016). AtNUDX23 has been reported to regulate flavin homeostasis and carotenoid biosynthesis associated with ROS scavenging in Arabidopsis via its FAD hydrolase activity (Maruta et al., 2012; Rao et al., 2024). In rice, OsNUDX2 mitigates oxidative stress by eliminating the oxidized nucleotides, specifically 8-oxo-dGTP, which are produced via ROS-induced DNA damage in rice (Kondo et al., 2022). This suggests that the function of NUDX2 in rice is related to ROS homeostasis maintenance. So far, the function of OsNUDX family remains largely unknown.

In this study, we investigated the physiological functions of OsNUDX23 during seed germination. Knockout of *OsNUDX23* resulted in accelerated coleoptile emergence and inhibited radicle elongation, both of which were attributed to elevated ROS levels. In addition, we observed a reduced germination rate and higher starch content in the seeds of *Osnudx23* mutants. Our findings demonstrated that OsNUDX23 plays a crucial role in maintaining optimal germination rates by modulating ROS level to prevent excessive ROS accumulation and by regulating starch metabolism in rice.

## Materials and methods

### Plant materials and growth conditions

The wild type (WT) *Oryza sativa* L. used in this study was Nipponbare (NIP), and various loss-of-function mutant lines *Osnudx23* and transgenic lines *proOsNUDX23::GUS* were generated in the NIP background. Rice plants were cultivated either under controlled laboratory conditions (32°C/28°C, 16h light/8h dark, 80% humidity) or in the paddy field at Shenzhen during the natural growing season. Seeds were dried for 14 days at 37°C prior to storage at 16°C.

### Identification of rice *NUDX* genes

We queried rice genes that exhibit similarity with Arabidopsis *NUDX* genes and possess a NUDIX motif (E<1e-5) using the BLAST program (https://tcoffee.crg.eu/apps/tcoffee/do:expresso) against rice genome database (RAP-DB https://rapdb.dna.affrc.go.jp). The HMM profile of NUDIX domain was retrieved from the Pfam database (http://pfam.xfam.org) and visualized using TBtools software (Chen et al., 2020). The amino acid sequences of *OsNUDX23* homologs across various species were aligned using SnapGene.

### Subcellular localization

To determine the subcellular localization of OsNUDX23, the full-length coding sequence was amplified and inserted into the p35S: GFP vector, which is driven by the 35S promoter, with GFP fused to the N-terminal of OsNUDX23. The recombinant construct was introduced into rice protoplasts via PEG-mediated transformation. Following 16 h incubation in darkness at room temperature, GFP fluorescence was examined using confocal laser scanning microscopy (Zeiss LSM 710). The empty vector served as a control.

### Enzymatic activity assays of OsNUDX23

To purify the OsNUDX23 protein *in vitro*, the 810 bp coding sequence of *OsNUDX23* was cloned into the pGEX 4T-1 vector using the ClonExpress^®^ II One Step Cloning Kit (Vazyme, C112). The recombinant plasmid was then introduced into the *Escherichia coli* strain BL21. Expression of the OsNUDX23-GST fusion protein was induced with 0.5 mM IPTG at 28°C for optimal expression. The OsNUDX23-GST protein was subsequently purified and enriched using a GST 4FF Pre-Packed Gravity Column (Sangon Biotech, C600911).

A method for the activity assay of Nudix hydrolases was used to determine enzymatic activity of OsNUDX23 (Ge et al., 2007). The enzymatic assays were conducted in a 50 μL reaction mixture containing 50 mM Tris-HCl (pH 8.5), 5 mM MgCl_2_, 1 mM dithiothreitol, 2 mM substrates, 4 unit of calf alkaline phosphatase and 5 μg of OsNUDX23 protein. Following 30-minute incubation at 37°C, the reaction was terminated by the addition 150 μL of 1 M H_2_SO_4_. Subsequently, 100 μL of water and 700 μL of freshly prepared mixture (0.42% molybdate. 4H_2_O: 10% ascorbic acid = 6: 1) were added. The reaction tubes were then incubated in a room temperature for 5 min to develop color, after which they were cooled to room temperature. Finally, the absorbance of the solutions was measured at 820 nm using a spectrophotometer. The reactions containing GST protein served as negative controls for each substrate.

### Generation of transgenic plants

Two target sites within the OsNUDX23 were selected for the design of guide RNA (gRNA) to facilitate RNA-guided genome editing (RGE) in rice. The gRNA cassette was integrated into the pHEC401 vector following the CRISPR-Cas9 method (Xie et al., 2014). Subsequently, the pHEC401-gRNA plasmid was then introduced into the *Agrobacterium* strain EHA105 and transformed into NIP rice embryogenic callus via a standard *Agrobacterium*-mediated transformation protocol. The genotypes of CRISPR/Cas9 plants were confirmed by sequencing analysis.

The 2 kb genomic sequence upstream of transcription start site of *OsNUDX23* was amplified and cloned into the pCAMBIA1301 vector with HindIII and NcoI restriction sites to generate the *proOsNUDX23::GUS* plasmid. The plasmid was then transformed into callus using *Agrobacterium tumefaciens* strain GV3101. The *proOsNUDX23::GUS* seeds were harvested from T0 transformants after confirmation. All primers used in this study were listed in **Supplementary Table S1**.

### Seed germination tests

Dried and dehulled seeds were surface-sterilized by immersing in 75% ethanol for 1 minute, followed by treatment with 30% (v/v) sodium hypochlorite solution for 30 minutes. Seeds were then washed with sterile double-distilled water seven times for 30 seconds each. One hundred seeds were placed on filter paper moistened with water and incubated at 30°C under a 16 h light/8 h dark photoperiod and 80% humidity for germination. Coleoptile elongation (≥1mm) was measured and counted at different imbibition times using a stereo microscope equipped with a digital camera (Leica S9D). Radicle emergence was considered the morphological marker indicating the completion of germination. Germination results are presented as mean ± SD values obtained from three independent replicates.

### GUS staining

Rice seeds were collected at various imbibition times and incubated in GUS staining buffer (50 mM NaH_2_PO_4_ pH 7.2, 50 mM Na_2_HPO_4_ pH 7.2, 10 mM Na_2_·EDTA, 0.1% Triton-X100, 0.5 mM K_3_Fe(CN)_6_, 2 mM X-Gluc, and 0.5 mM K_4_Fe(CN)_6_). The samples were subjected to vacuum infiltration 3–5 times. Subsequently, the samples were incubated at 37°C for overnight. After staining, the samples were washed with 70% ethanol at room temperature. Images were captured by a stereo microscope (Leica S9D).

### RNA-seq analysis

Total RNA was extracted from WT and *Osnudx23* seeds at 0 h, 12 h, and 24 h post-imbibition and used for library construction with the Illumina TruseqTM RNA sample prep Kit. Subsequently, RNA sequencing was performed on the Illumina NovaSeq 6000 platform. The resulting reads were mapped to the *Oryza sativa* L. genome (IRGSP-1.0) and gene expression levels were quantified and normalized to reads per kilobase million (RPKM). Differentially expressed genes (DEGs) were identified using DEseq2, with a threshold of p-value < 0.05 and |log_2_(fold change)| ≥ 1. The expression profiles of selected DEGs during seed germination were visualized using tools available on Omicshare (https://www.omicshare.com/tools/).

### Quantitative real-time PCR

Total RNA was extracted from the samples using TRIzol Reagent (Ambion, 155960-18), followed by the synthesis of the first-strand cDNA using HiScript III RT SuperMix for qPCR (+gDNA wiper) (Vazyme, R323). RT-qPCR was performed on a CFX96TM Real-Time System (BIO-RAD, CFX96) using ChamQ Universal SYBR qPCR Master Mix (Vazyme, Q711). Each sample included at least three biological replicates, each with three technical replicates. The rice actin served as an internal control. The relative expression levels were calculated using the comparative 2^−ΔΔCt^ method (Livak and Schmittgen, 2001), and the primer sequences are listed in the **Supplementary Table S1**.

### H_2_O_2_ and starch contents quantification

The H_2_O_2,_ starch, and sucrose contents were detected following to the instructions provided in the H_2_O_2_ Content Assay Kit (mlbio, mlsh0699), Starch Content Assay Kit (Solarbio, BC0700), and Plant Sucrose Content Assay Kit (Solarbio, BC2465), respectively. Other assay kits, such as Plant FAD Elisa Assay Kit (kehansheng, KHS-161156K) for FAD content, NAD(H) Content Assay Kit (Solarbio, BC0310) for NAD^+^ content, NADP(H) Content Assay Kit (MTT) (Solarbio, BC1100) for NADP(H) content, NADH Oxidase (NOX) Activity Assay Kit (Solarbio, BC0630) for NOX activity, Total Antioxidant Capacity (T-AOC) Assay Kit (Solarbio, BC1315) for total antioxidant capacity.

## Results

### Identification and characterization of *OsNUDX*23 in rice

**Figure 1:**
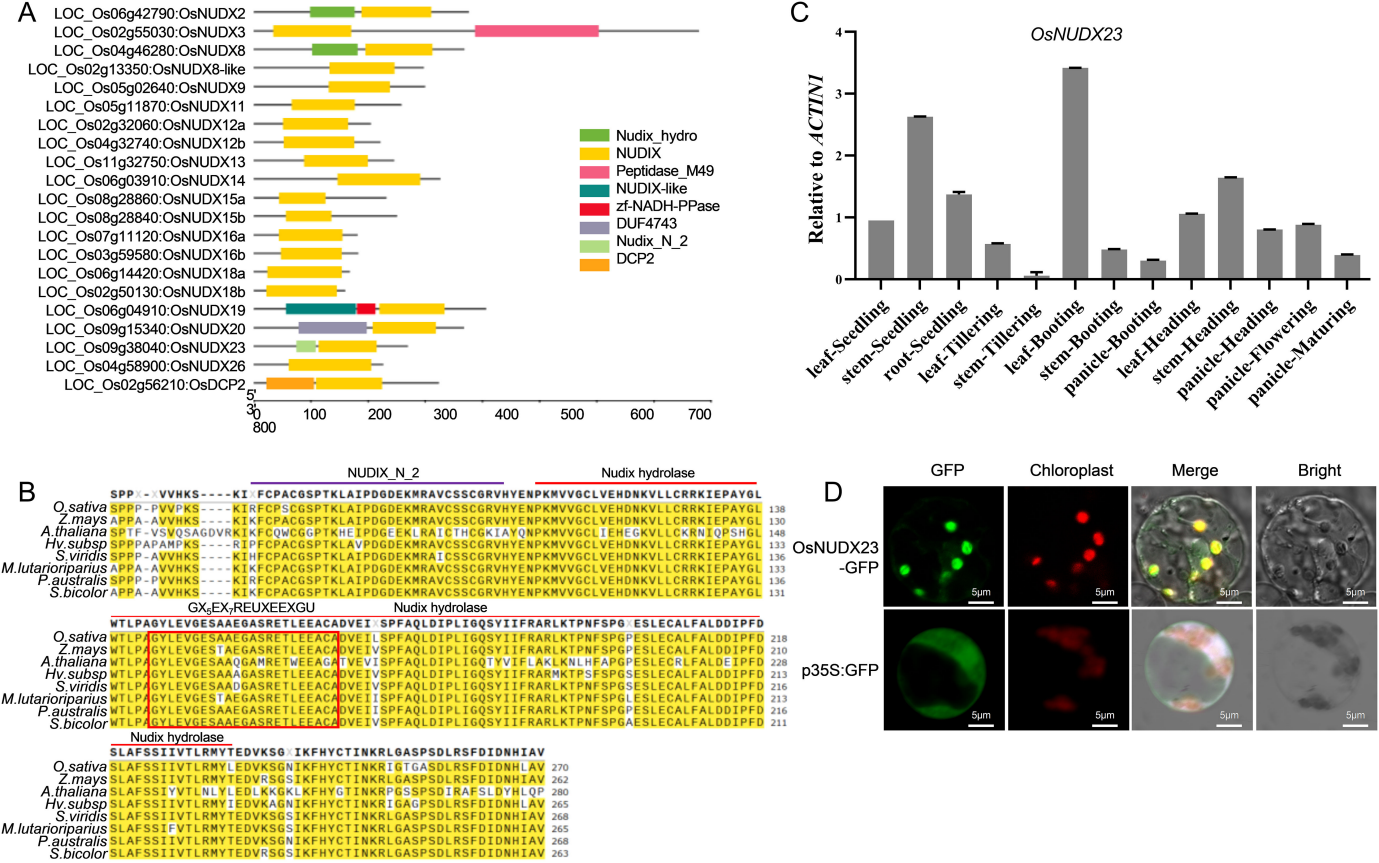
Characteristics and expression profile of *OsNUDX23.* **(A)** The conserved canonical NUDIX domain (highlighted in yellow) and additional predicted domains in the OsNUDX proteins. **(B)** Alignment of partial amino acid sequences among the orthologs of OsNUDX23. **(C)**The expression patterns of OsNUDX23 in various organs. **(D)** The subcellular localization of OsNUDX23 in rice protoplast. Scale bar: 3µm.

To conduct a comprehensive analysis of the *NUDX* gene family in the rice genome, we performed a BLAST search against the conserved NUDIX hydrolase domain (PF00293) within the Rice Annotation Project Database (RAP-DB, https://rapdb.dna.affrc.go.jp) and a conserved domain search in the National Center for Biotechnology Information (NCBI, https://www.ncbi.nlm.nih.gov/). A total of 21 putative OsNUDX-encoding genes were identified, and their protein sequences along with annotation files (IRGSP-1.0) were retrieved for further domain alignment analysis. In addition to the canonical NUDIX hydrolase domain present in all predicted OsNUDX proteins, several OsNUDX members also contain additional domains such as NUDIX-like, peptidase_M49, NADH-PPase, DUF4743, DCP2 and Nudix_N_2 domains (**Figure 1A**). The *OsNUDX23* (LOC_Os09g38040)-encoded protein contains both the canonical NUDIX hydrolase domain and the Nudix_N_2 domain. To investigate the conservation of these domains across plant species, we collected the amino acid sequences of the homologous proteins of OsNUDX23 from *Zea mays*, *Arabidopsis thaliana*, *Hordeum vulgare*, *Setaria viridis*, *Miscanthus lutarioriparius*, *Phragmites australis*, and *Sorghum bicolor* for sequence alignment analysis. All identified NUDX23 proteins possess both the Nudix_N_2 domain and the NUDIX hydrolase domain. Additionally, many residues within the GX5EX7REUXEEXGU motif (where U represents I, L, or V) at the active sites of NUDIX hydrolases reveal highly conservation among graminaceous crops but exhibit lower conservation in the dicotyledonous Arabidopsis (**Figure 1B**).

In Arabidopsis, AtNUDX23 has been reported to regulate flavin homeostasis and carotenoid biosynthesis (Maruta et al., 2012; Rao et al., 2024). However, the function of OsNUDX23 in rice remains uncharacterized. To elucidate the potential role of *OsNUDX23*, we examined its expression patterns across various tissues, including leaves, roots, stems and panicles using RT-qPCR. Our results demonstrated that *OsNUDX23* is expressed in nearly all tested tissues, with the highest levels observed in leaves during booting stage, followed by stem tissue in seedlings. In contrast, the expression level of *OsNUDX23* was relatively low in tillering and maturing stage, as well as in stems and panicles during the booting stage (**Figure 1C**). This widespread expression pattern suggests that *OsNUDX23* may play diverse physiological roles in rice. Moreover, the NUDX proteins have been classified into three groups based on their predicted subcellular localizations: cytosol, mitochondrion and chloroplast, and AtNUDX23 has been shown to localize in chloroplasts (Ogawa et al., 2008). To elucidate the subcellular localization of OsNUDX23, a transient expression vector encoding the OsNUDX23-GFP fusion protein was constructed and transiently introduced into rice protoplasts. The GFP fluorescence signals were observed to colocalize with chlorophyll autofluorescence, indicating that OsNUDX23 is localized within chloroplasts (**Figure 1D**).

### OsNUDX23 acts as an active Nudix hydrolase

**Figure 2:**
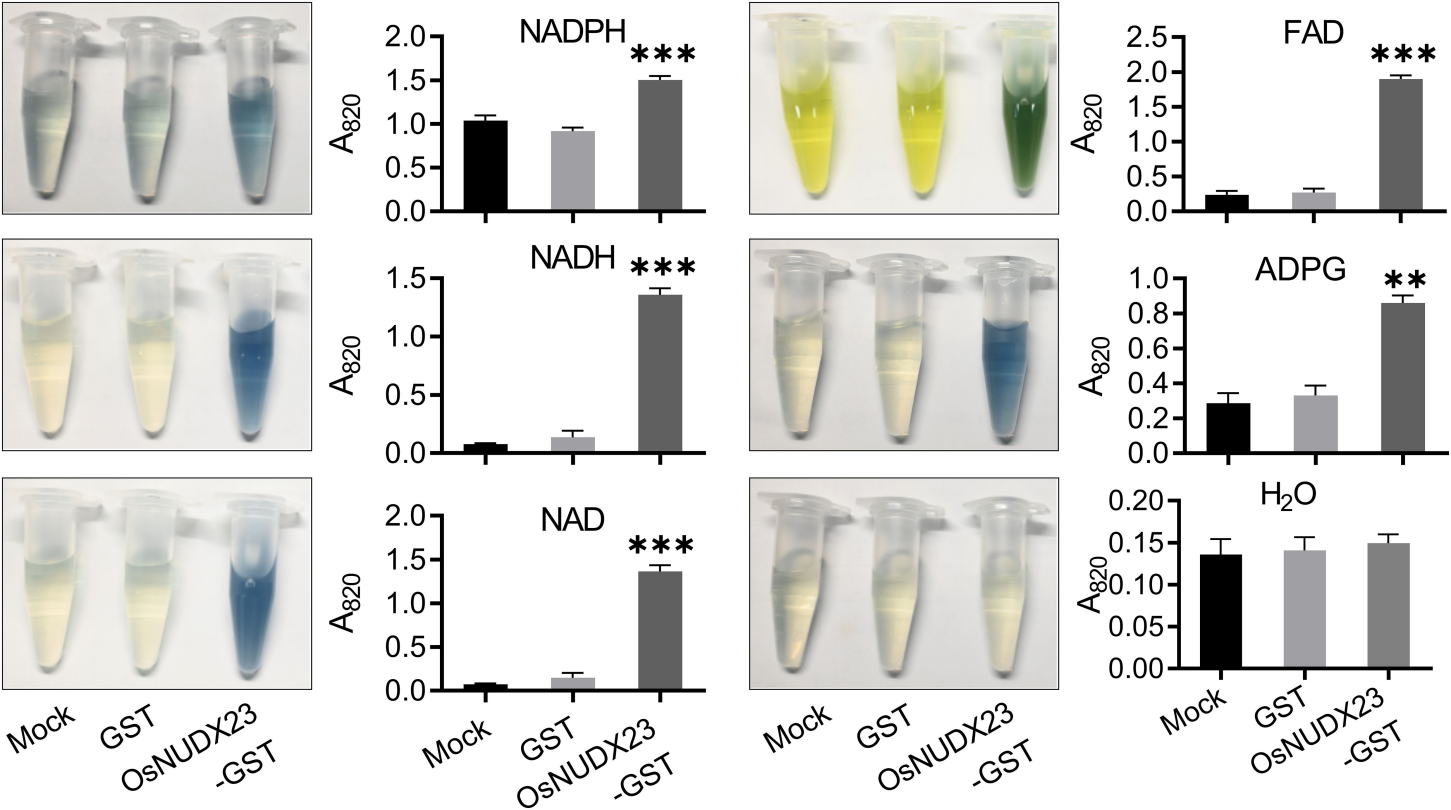
OsNUDX23 functions as a nucleoside diphosphate hydrolase and exhibits hydrolytic activity towards various nucleotides. Mock treatment and GST protein were used as negative controls, and H_2_O replaced the substrate for the blank control. Enzymatic activity was quantified by measuring absorbance at 820 nm (**p < 0.01, ***p < 0.001).

The Nudix family, renowned for its catalytic function in hydrolyzing nucleoside diphosphates linked to various moieties (denoted as X), plays a crucial role in cellular metabolism (Bessman et al., 1996). In Arabidopsis, AtNUDX23 negatively regulates flavin homeostasis via hydrolyzing FAD (Maruta et al., 2012). To determine whether OsNUDX23 exhibits Nudix hydrolases activity, we expressed the GST-OsNUDX23 fusion protein in *E. coli* and purified it for incubation with various nucleoside diphosphate-X substrates. Upon hydrolysis of the substrates by OsNUDX23, the inorganic phosphate product was quantified colorimetrically (Ge et al., 2007). The results demonstrated that GST-OsNUDX23 exhibited robust hydrolytic activity towards NADPH, NADH, NAD, FAD and ADPG, whereas the GST control displayed no enzymatic activity (**Figure 2**).

Given that most Nudix hydrolases require divalent metal ions, such as Mg^2+^, Mn^2+^ and Ca^2+^, along with appropriate pH and temperature conditions (Ito et al., 2012), we further characterized the enzymatic activity of OsNUDX23 by investigating its optimal ionic conditions, pH, and temperature (**Supplementary Figure S1**). In reactions using NADH as the substrate, supplementation of 5 mM MgCl_2_ or MnCl_2_ resulted in detectable activity of OsNUDX23, while CaCl_2_ or the absence of these ions did not support its activity (**Supplementary Figure S1A**). The optimal pH for OsNUDX23 activity was determined to be 9.0 in Tris-HCl (pH6.5-9.0) and 9.5 in glycine-NaOH (pH8.5-10.0) buffers (**Supplementary Figure S1B**). Furthermore, the enzyme exhibited maximal activity at 50°C among the tested temperature conditions ranging from 25°C to 60°C (**Supplementary Figure S1C**). Collectively, these findings indicate that OsNUDX23 functions as a nucleoside diphosphate hydrolase under the favorable environmental conditions.

### OsNUDX23 affects the emergence of rice coleoptile during early germination

**Figure 3:**
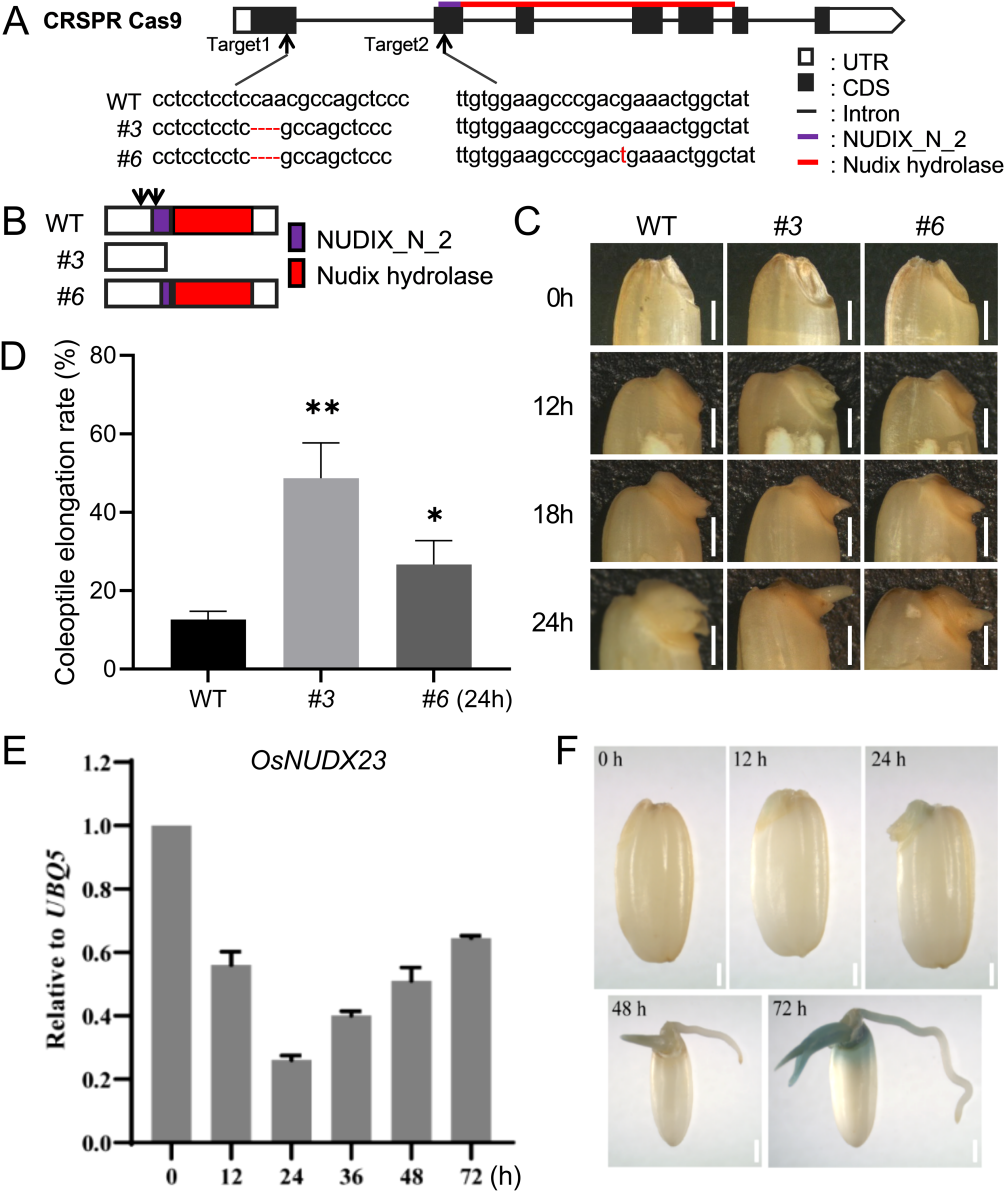
*OsNUDX23* affects seed coleoptile elongation in rice. **(A)** Schematic diagram of the *OsNUDX23* gene structure and two guide RNA target sites. **(B)** Schematic representation of the encoded proteins in Osnudx233 and Osnudx236 mutant lines compared to WT. **(C)** The phenotype of coleoptiles in WT and Osnudx23 lines seeds within 24 h post-imbibition. Scale bar: 1mm. **(D)** Coleoptile elongation rate of WT and *Osnudx23* lines seeds at 24 h post-imbibition time. The length of coleoptile is ≥1mm. Data represent mean ± SD from three biological replicates, each with 100 seeds. Statistical significance was determined by two-sided Student’s t test (*p < 0.05, **p < 0.01). **(E)** Expression levels of *OsNUDX23* over a 72 h post-imbibition. **(F)** GUS expression driven by the *OsNUDX23* promoter (*proOsNUDX23::GUS*) was observed from 0 to 72 h post-imbibition. Scale bar: 2mm.

To explore the biological role of *OsNUDX23* in rice development, we generated the mutant lines of *OsNUDX23* using the CRISPR/Cas9 genome-editing approach in the *Japonica* Nipponbare (NIP) variety. Two target sites were selected within the first and second exons of *OsNUDX23*, respectively (**Figure 3A**). In the T_2_ generation, we successfully obtained two independent *Osnudx23* mutant lines and confirmed the mutations by sequencing (**Figure 3A**). Notably, the *Osnudx23#3* line exhibited a 4-bp deletion, resulting in a premature stop codon in *OsNUDX23*, while the *Osnudx23#6* line displayed a structural alteration in the NUDIX_N_2 domain due to the 4-bp deletion and 1-bp insertion (**Figures 3A, B**). These two homozygous *Osnudx23* lines were selected for further characterization. No significant phenotypic differences were observed between *Osnudx23* lines and the wild-type (WT) at the seedling stage (**Supplementary Figure S2A**). However, both *Osnudx23#3* and *Osnudx23#6* lines showed delayed radicle growth compared to the WT during seed germination stage (**Supplementary Figure S2B**), indicating that OsNUDX23 may participate in regulating seed germination.

Further investigation revealed significant phenotypic changes in both *Osnudx23* mutants at the early stage of seed germination. Specifically, the two *Osnudx23* lines showed earlier coleoptile emergence compared to WT, with noticeable differences observed as early as 24 h post-imbibition (**Figure 3C**). The percentage of WT seeds showing coleoptile elongation (≥1mm) was 13% at 24 h post-imbibition, whereas both *Osnudx23#3* and *Osnudx23#6* lines showed a rapid increase in coleoptile elongation rate, reaching 49% and 27%, respectively (**Figure 3D**). To validate this phenotype, we examined the coleoptile elongation rate of two additional mutant lines: *Osnudx23#2*, which harbors a 5-bp deletion at target 1, and *Osnudx23#22*, which contains a combination of 1-bp insertion at target 1 and a 5-bp deletion at target 2, leading to premature termination of OsNUDX23 (**Supplementary Figures S3A, B**). All *Osnudx23* lines consistently displayed accelerated coleoptile emergence compared to WT during the germination stage (**Supplementary Figures S3C, D**). However, while 98% of the WT rice seeds exhibited coleoptiles elongated ≥1 mm by 30 h post-imbibition, this percentage ranged from 60% to 90% in the *Osnudx23* lines (**Supplementary Figure S3E**). These findings implied that the disruption of *OsNUDX23* promotes early coleoptile emergence but may reduce the overall seed germination rate.

Interestingly, in contrast to the growth of coleoptile, all *Osnudx23* lines exhibited a delay in radicle elongation following 30 h of imbibition compared to WT (**Supplementary Figure S4A**). In WT, the percentage of seeds with radicle elongation (≥1 mm) increased rapidly, reaching 48% at 36 h and 95% at 72 h post-imbibition. However, in the *Osnudx23* lines, radicle growth was markedly inhibited, with only 4-16% seeds showing radicle elongation (≥1 mm) at 36 h, and 75-85% at 72 h, both significantly lower than WT (**Supplementary Figure S4B**). Statistical analysis confirmed that the percentage of coleoptile elongation prior to radicle emergence was significantly higher in all *Osnudx23* lines than that in WT at 24 h post-imbibition (**Supplementary Figure S3D**). These results indicate that *OsNUDX23* may exhibit differential effects during early and later stages of seed germination, particularly influencing the growth dynamics of the coleoptile and radicle.

To elucidate the interplay between *OsNUDX23* and seed germination, we investigated the expression profile of *OsNUDX23* during this process. RT-qPCR analysis was performed to examine the transcript levels of *OsNUDX23* over a 72 h period post-imbibition. The results revealed a significant decline in *OsNUDX23* transcript levels from 0 to 24 h post-imbibition, followed by a consistent increase from 24 h to 72 h, with the lowest level at 24 h (**Figure 3E**). Additionally, we generated the transgenic plants using the *OsNUDX23* promoter to drive *GUS* expression. GUS staining revealed that the promoter activity commenced at 24 h post-imbibition (**Figure 3F**). By 72 h post-imbibition, strong GUS signals were detected around the seed embryo, as well as in the coleoptile and elongating plumule, while weaker staining was observed in the radicle near the embryo (**Figure 3F**). The absence of signal at 0 h is likely due to the limited penetration of the staining reagent into the non-imbibed seed. Collectively, these temporal and spatial expression pattens suggest a potential regulatory role of OsNUDX23 during the early stages of rice seed germination.

### The disruption of *OsNUDX23* leads to alterations in ROS metabolic pathways

To elucidate the role and uncover the molecular mechanisms underlying rice seed germination regulated by *OsNUDX23*, we conducted RNA-seq analyses on WT and *Osnudx23#3* mutant seeds at 0, 12 and 24 h post-imbibition. A substantial number of raw reads, ranging from 23 to 33 million, were obtained, and a total of 25,670 genes were successfully detected and mapped to the rice genome (IRGSP-1.0). The differentially expressed genes (DEGs) between *Osnudx23* and WT at different time points were identified using the DESeq2 package, with a false discovery rate (FDR) < 0.05 and |log2 (fold change)|>1 as thresholds.

**Figure 4:**
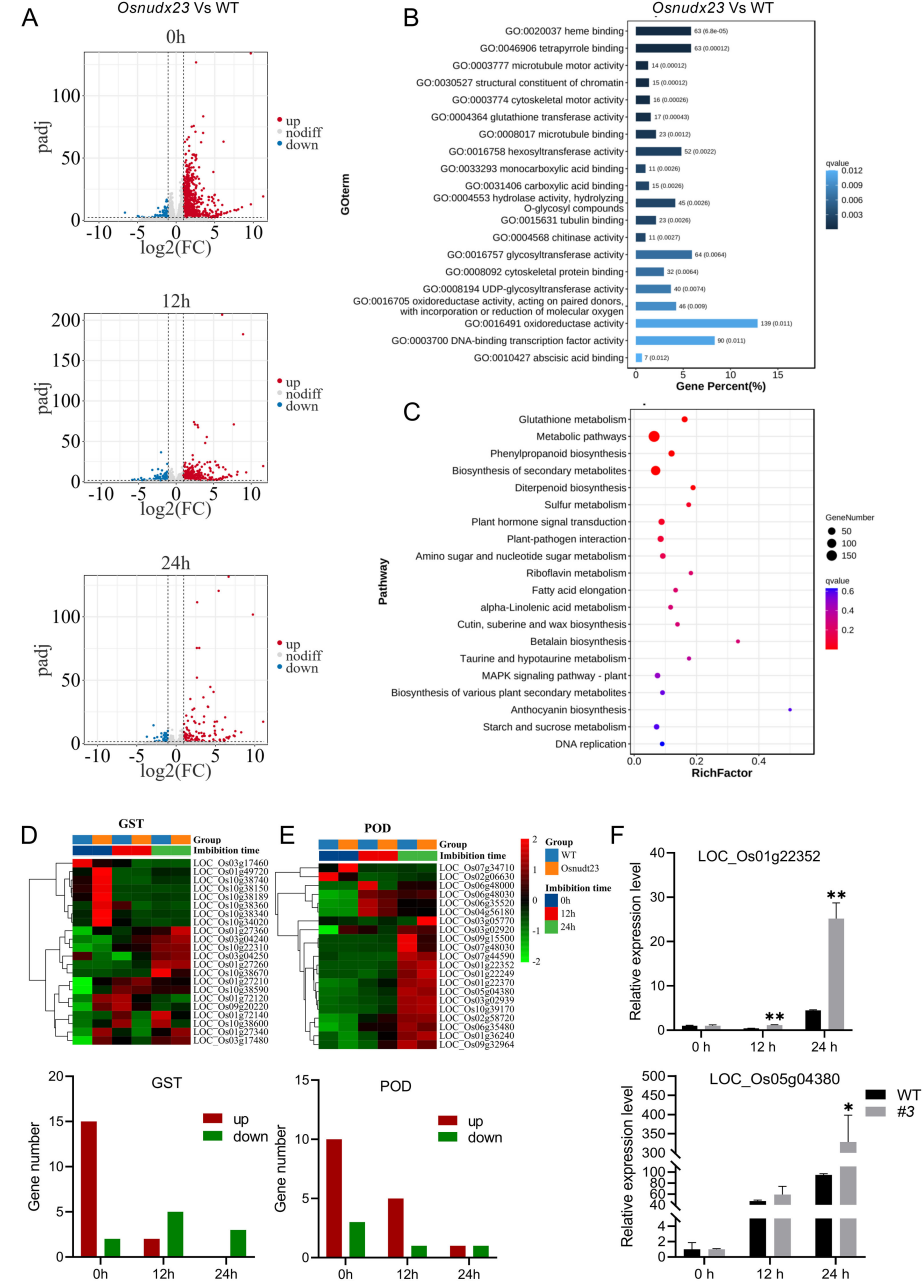
Transcriptome analysis of wild-type and *Osnudx23* at germination stage. **(A)** Volcano plot showing DEGs in Osnudx233 compared to WT seeds at various time points. **(B)** Top 20 over-represented Gene Ontology (GO) biological process among DEGs in *Osnudx23.* **(C)** Top 20 the Kyoto Encyclopedia of Genes and Genomes (KEGG) pathways enriched by all up-regulated DEGs involved in seed germination. **(D, E)** Heatmaps illustrating expression patterns of 22 DEGs encoding GST **(D)** and 21 DEGs encoding POD **(E)** in nudx233 line relative to WT. **(F)** Expression analysis of *LOC_Os01g22352* and *LOC_Os05g04380* over time after imbibition in WT and *Osnudx23.* Values are mean ± SD (n = 3). *p < 0.05, **p < 0.01.

To investigate the changes in gene expression in response to OsNUDX23, we analyzed the DEGs at 0, 12 and 24 h post-imbibition in *Osnudx23#3* relative to WT. Specifically, 1,583 DEGs were identified in *Osnudx23#3* line compared to WT at 0 h, including 1,225 upregulated and 358 downregulated genes. At 12 h post-imbibition, 795 DEGs (628 upregulated and 167 downregulated) were observed, while at 24 h, only 194 DEGs (134 upregulated and 60 downregulated) were detected (**Figure 4A**). Gene Ontology (GO) analysis revealed significant enrichment in several molecular function categories, including heme binding, tetrapyrrole binding, oxidoreductase activity, glycosyltransferase activity, hexosyltransferase activity, hydrolase activity, and DNA-binding transcription factor activity (**Figure 4B**). KEGG pathway analysis indicated that the most significantly enriched pathways were primarily associated with glutathione metabolism and starch metabolism (**Figure 4C**). Notably, a substantial number of POD and glutathione S-transferase (GST) genes were enriched in these metabolic pathways. Further refinement identified 22 DEGs encoding GST and 21 DEGs encoding POD (**Figures 4D, E**), both of which are involved in ROS scavenging in cells (Gomes and Garcia, 2013). Interestingly, some *GST* genes exhibited highest expression levels at 0 h post-imbibition and the levels gradually decrease at 12 h and 24 h in *Osnudx23*, while an increased expression trend was observed in WT (**Figure 4D**). Two genes (*LOC_Os01g22352*, *LOC_Os05g04380*) were selected for qPCR analysis during seed germination, and the expression trend was consistent with the RNA-seq data (**Figure 4F**). These results indicate that *OsNUDX23* may participate in regulating rice seed germination through the ROS metabolic pathway.

### *OsNUDX23* mitigates ROS accumulation during seed germination

**Figure 5:**
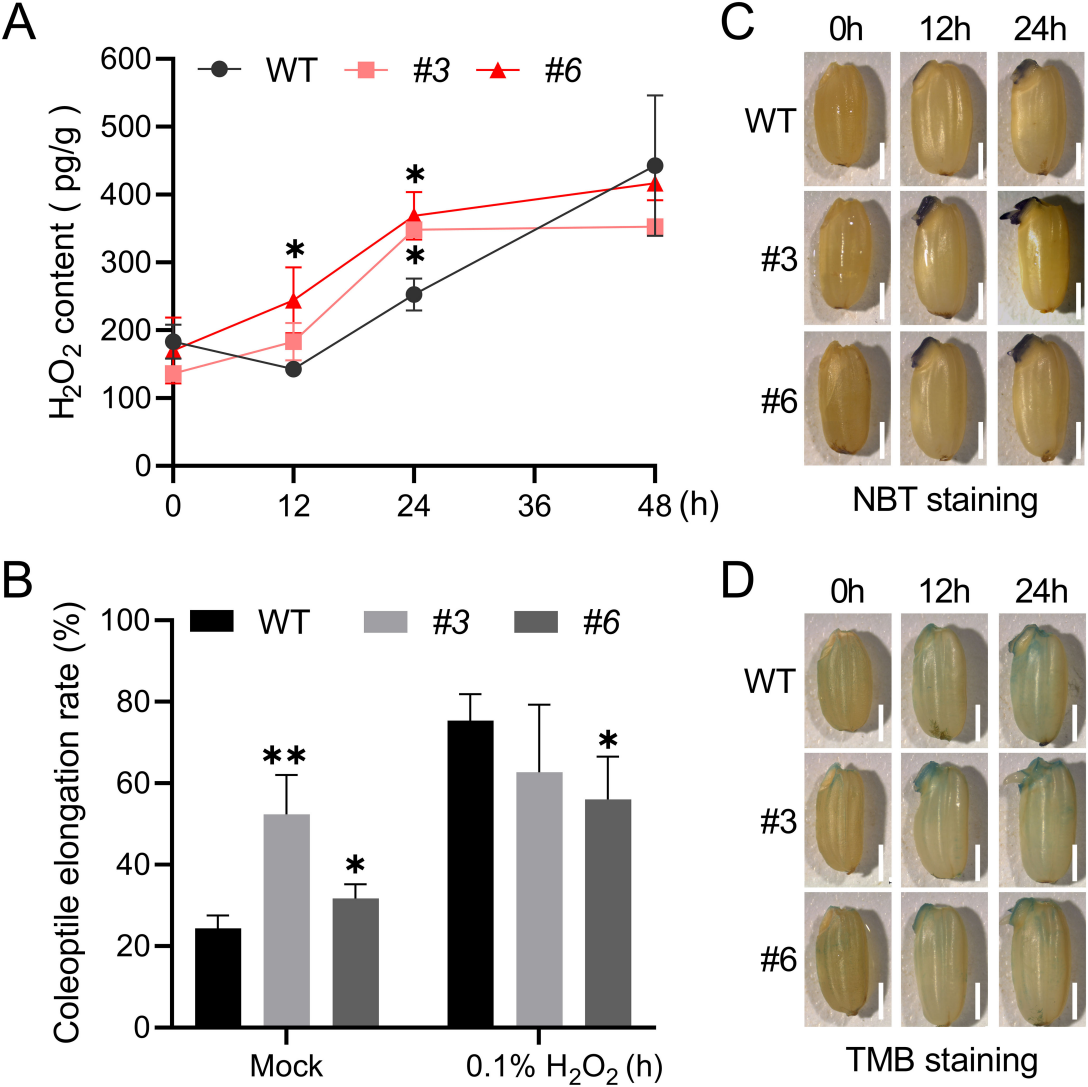
OsNUDX23 mitigates ROS accumulation during seed germination. **(A)** Quantification of H_2_O_2_ content in seeds of WT and *Osnudx23* lines at different time point. Data represent the mean ± SD from three biological replicates, each consisting of 20 germinating seeds. **(B)** The coleoptile elongation rate of WT and *Osnudx23* lines exposed to 0.1% H_2_O_2_. Data are presented as the mean ± SD from three biological replicates, with 100 seeds per replicate. Significance was determined by two-sided Student’s t test (*p < 0.05, **p < 0.01). **(C, D)** Histochemical staining by nitro blue tetrazolium (NBT) and 3,3’,5,5’-Tetramethylbenzidine (TMB) to visualize the content of O_2_^-^ and ·OH in WT and *Osnudx23* lines. Scale bar: 2mm.

ROS, including O_2_^-^, H_2_O_2_ and ·OH, play a dual role in seed germination and signal transduction which only occur when the ROS concentration falls into an “oxidative window” (Bailly et al., 2008). To ascertain the impact of *OsNUDX23* on ROS accumulation, we measured endogenous H_2_O_2_ levels in WT and *Osnudx23* lines (*Osnudx23#3* and *Osnudx23#6*) at 0, 12, 24 and 48 h post-imbibition. The *Osnudx23#3* and *Osnudx23#6* seeds exhibited consistently higher H_2_O_2_ content compared to WT, with significant increases observed at 12 and 24 h (**Figure 5A**). However, by 48 h post-imbibition, no significant difference in H_2_O_2_ levels was detected between WT and *Osnudx23* lines (**Figure 5A**). These results suggest that OsNUDX23 plays an important role in regulating H_2_O_2_ accumulation during the early stage of seed germination.

To determine whether H_2_O_2_ accumulation affects coleoptile elongation, we subjected seeds to exogenous H_2_O_2_ treatment during seed germination. The results demonstrated that the percentage of coleoptile elongation ≥1 mm in both WT and *Osnudx23* mutants increased at 24 h post-imbibition when exposed to 0.1% H_2_O_2_. Notably, the coleoptile elongation percentage was pronounced in the WT and *Osnudx23#6* lines under 0.1% H_2_O_2_ treatment compared to the water control group (**Figure 5B**). However, this increase was attenuated in *Osnudx23#3* line (**Figure 5B**), suggesting that low concentration of H_2_O_2_ accumulation can promote the coleoptile elongation, while the elevated H_2_O_2_ levels in *Osnudx23* mutants may diminish this effect. Conversely, exposure to 0.5% H_2_O_2_ resulted in only minor changes in the coleoptile elongation for WT seeds compared to the control group, but it significantly inhibited in *Osnudx23#3* (**Supplementary Figure S5**), indicating that excessive accumulation of H_2_O_2_ inhibits coleoptile growth. Collectively, these findings suggest that the loss of *OsNUDX23* leads to H_2_O_2_ accumulation, which promotes seed germination at low concentrations but inhibits it when accumulated excessively.

Other types of ROS, such as O_2_^-^ and ·OH, can break dormancy and directly mediate cell wall loosening during seed germination (Müller et al., 2009; Oracz et al., 2009). Hence, we examined the production of O_2_^-^ and ·OH in rice seeds at various imbibition times by histochemical staining. Strong nitroblue tetrazolium (NBT) staining was observed in the coleorhiza of *Osnudx23* mutant lines at 12 and 24 h, while no significant staining was detected in the WT coleoptile (**Figure 5C**). This suggests that O_2_^-^ may play a distinct role in protrusion of coleoptile and coleorhiza. ·OH can be formed from O_2_^-^ and H_2_O_2_ in the apoplast under catalysis by peroxidases (Schweikert et al., 2002). Given that ·OH has the shortest lifespan among ROS, direct quantification is challenging (Müller et al., 2009). Therefore, we measured the peroxidase activity as an indirect indicator of ·OH production (Zhang et al., 2014). Peroxidase activity in rice seeds was determined by 3,3’,5,5’-Tetramethylbenzidine (TMB) staining, which revealed increased intensity in the embryo of *Osnudx23* mutant lines at 12 h compared to WT, particularly in the coleorhiza. It also showed staining in the coleorhiza of WT and *Osnudx23* mutant lines at 24 h (**Figure 5D**). These findings indicate that ROS accumulation may be higher in *Osnudx23* mutant lines, promoting coleoptile elongation at 12 and 24 h. Collectively, these results suggest that OsNUDX23 inhibits ROS levels during seed germination.

### *OsNUDX23* inhibits ROS accumulated by regulating NADP/NADPH ratio

**Figure 6:**
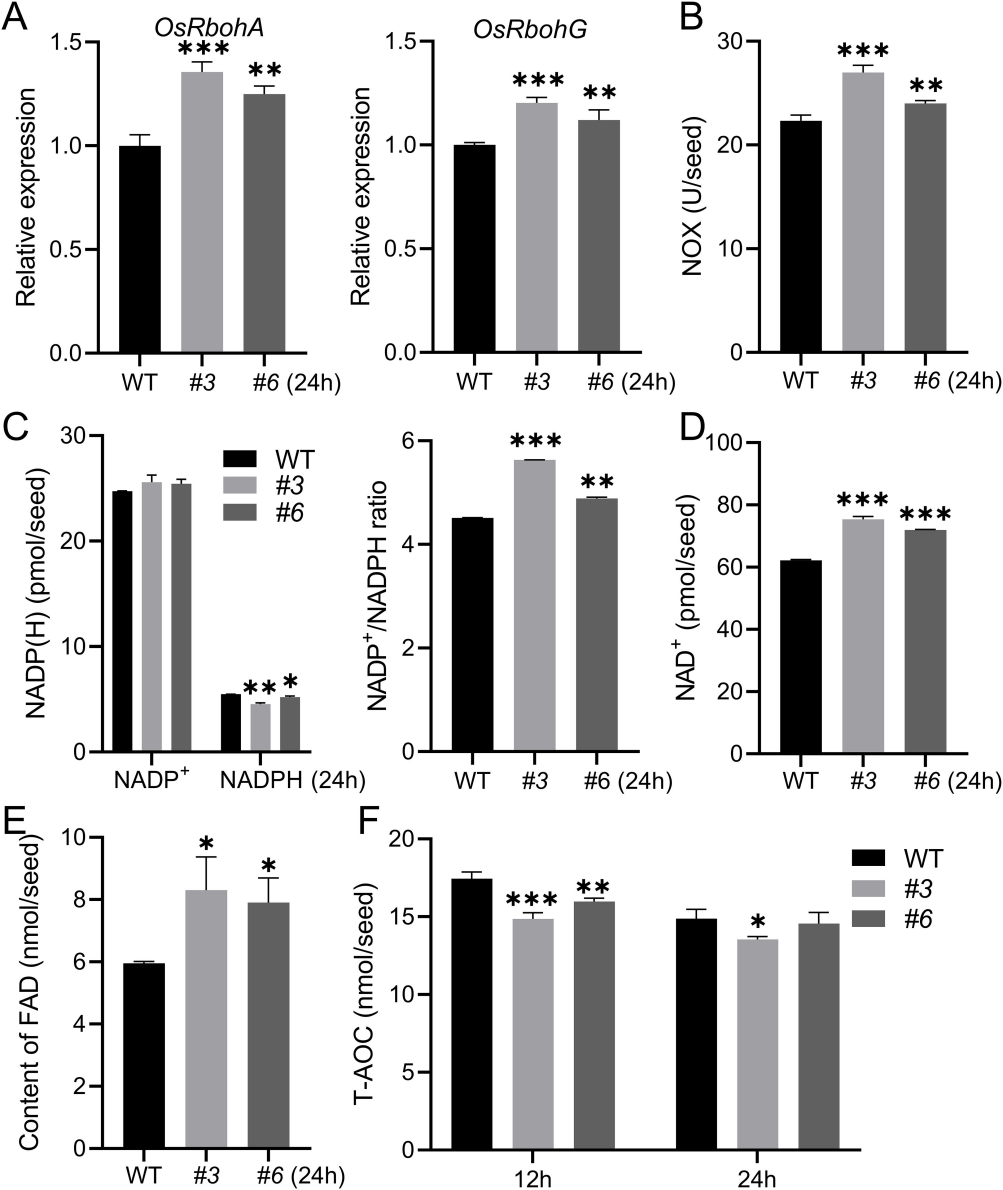
OsNUDX23 influences NOX activity and antioxidant enzyme system. **(A)** Expression analysis of *OsRbohA* and *OsRbohG* in WT and *Osnudx23* seeds after 24 h imbibition. **(B)** NADPH oxidase activities in WT and *Osnudx23* after 24 h imbibition. **(C)** NADP(H) contents and NADP^+^/NADPH Ratio in WT and *Osnudx23* seeds after 24 h imbibition. **(D, E)** The content of NAD^+^ and FAD. **(F)** Changes in T-AOC at 12 h and 24 h All data are means ± SD (n = 3), *p < 0.05, **p < 0.01, ***p < 0.001.

The plasma membrane NADPH oxidases (NOXs), also known as respiratory burst oxidase homologs (RBOHs), are the primary producers of ROS in a NADPH-dependent manner during various developmental stages in plants (Hu et al., 2020; Suzuki et al., 2011). Nine genes encoding RBOHs have been identified and designated as OsRbohA-I or OsNOX1-9 (Wang et al., 2013; Wong et al., 2007). To investigate whether the elevated accumulation of ROS in *Osnudx23* mutants is resulted from the regulation of NOXs, we examined the transcriptional levels of *OsRBOH* genes in WT and *Osnudx23* mutant plants. The results showed that the expression of *OsRbohA* and *OsRbohG* was upregulated in *Osnudx23* plants at 24 h compared to WT (**Figure 6A**). Furthermore, NOX activities were assessed, revealing higher activities in *Osnudx23* mutants compared to WT (**Figure 6B**). To investigate the role of NOX activity in coleoptile elongation, rice seeds were incubated in water or water containing the NOX-specific inhibitor DPI at concentrations of 12.5 μM and 25 μM. The results demonstrated that DPI treatment significantly inhibited the rapid elongation of the coleoptile phenomenon in *Osnudx23* mutant at 24 h (**Supplementary Figure S6**), highlighting the role of NOX activity in ROS-dependent coleoptile elongation. Tn addition, the levels of NADP^+^ were markedly higher than those of NADPH, and the NADP^+^/NADPH ratio was elevated in *Osnudx23* lines compared to WT at 24 h (**Figure 6C**), consistent with the elevated ROS levels observed in *Osnudx23* (**Figures 5A, C, D**). NAD is a coenzyme involved in catabolic pathways and acts as the electron acceptor in biosynthetic processes (Kramer et al., 2004; Pétriacq et al., 2013). The balance between NADP^+^ and NADPH, as well as NAD^+^ and NADH, is crucial for cell redox reactions that regulate germination initiation and plant growth (Hunt and Gray, 2009). Notably, NAD^+^ levels in *Osnudx23* seeds were markedly elevated (**Figure 6D**). These findings suggest that disruption of *OsNUDX23* enhances the activity of NOX and alters the ratios of NADP^+^/NADPH and NAD^+^/NADH, subsequently leading to increased ROS accumulation and accelerated coleoptile elongation.

FAD serves as a crucial coenzyme that links NADPH and oxygen molecules, acting as an electron carrier to transfer electrons from cytosolic NADPH to extracellular O_2_, thereby generating O^2-^ (Sagi and Fluhr, 2006). To investigate the effect of OsNUDX23 on the levels of ROS during seed germination, we quantified various substance contents in both WT and *Osnudx23* mutants. Notably, FAD levels were significantly elevated in *Osnudx23* at 24 h (**Figure 6E**), suggesting that OsNUDX23 plays a regulatory role in modulating cellular redox levels. Furthermore, our results indicated that OsNUDX23 may influence the cellular redox state (**Figure 4**). To further validate this hypothesis, we assessed the total antioxidant capacity (T-AOC), which serves as a key indicator for evaluating the overall antioxidant defense system and reflects the efficiency of the plant’s antioxidant enzyme system (Zhang et al., 2012). Our data demonstrated that WT exhibited significantly higher T-AOC levels at both 12 h and 24 h post-germination (**Figure 6F**), implying that OsNUDX23 contributes to mitigating cellular oxidation stress.

### *OsNUDX23* influences starch metabolism and development

**Figure 7:**
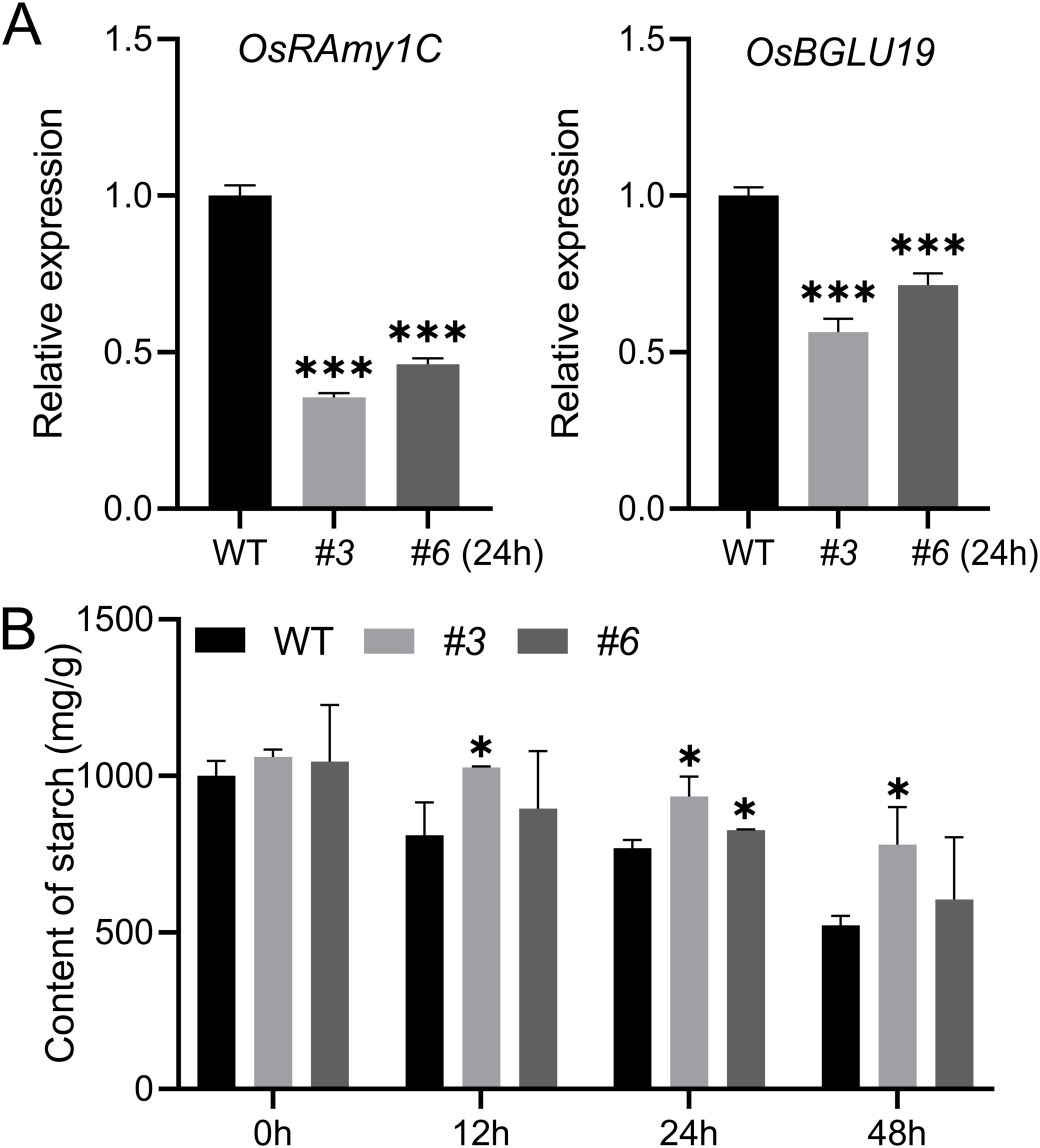
OsNUDX23 regulates starch metabolism. **(A)** Expression levels of *OsRAmy3A* and *OsBGLU19* after 24 h of imbibition. Values are means ± SD (n = 3), ***p < 0.001. **(B)** Quantification of starch content over time during germination. Data represent the mean ± SD from three biological replicates. Significance was determined by two-sided Student’s t test, *p < 0.05.

Starch reserve breakdown is essential for supplying energy and carbon skeletons required for embryonic development (Damaris et al., 2019). The enzymatic activity assays revealed that OsNUDX23 exhibited activity on ADPG (**Figure 2**), which serves as the initial substrate of starch synthesis, indicating that OsNUDX23 may play a role in starch metabolism. In addition, the expression levels of genes involved in starch and sucrose metabolism, such as *OsRAmy3A* and *OsBGLU19*, were down-regulated at 24 h post-imbibition in *Osnudx23* plants compared to WT (**Figure 7A**). Starch contents were measured in seeds at 0, 12, 24, and 48 h post-imbibition, revealing elevated starch accumulation in *Osnudx23#3* than WT at all tested timepoints (**Figure 7B**). In addition, the *Osnudx23* mutant exhibits significantly lower degradation rate of starch during post-imbibition compared to WT (**Supplementary Figure S7**). These findings collectively indicate that OsNUDX23 likely participates in starch degradation.

## Discussion

### OsNUDX23 modulates the physiological state of seed germination via hydrolyzing specific substrates

NUDXs, known as Nudix hydrolases, are ubiquitous across various species and are proposed to serve a “housekeeping” function by eliminating excess toxic metabolites or regulating the availability of intermediates in metabolic pathways (McLennan, 2006). In Arabidopsis, the NUDX family proteins are localized in various subcellular compartments including the cytosol, mitochondria, and chloroplasts (Kraszewska, 2008; Ogawa et al., 2005, 2008), and have been shown to be able to hydrolyze diverse nucleoside diphosphate derivatives *in vitro* (Ogawa et al., 2008). OsNUDX23 exhibits NAD, NADH, NADPH, ADPG and FAD pyrophosphohydrolase activities (**Figure 2**), playing an important role in the cellular energy metabolism. NAD and NADH are essential coenzymes involved in the electron transport chain, providing energy for various cellular physiological activities (Schertl and Braun, 2014; Smith et al., 2021). NADPH also functions as an electron donor in redox reactions (Hu et al., 2020). Starch generates energy via amylase-mediated hydrolysis, while ADPG serves as the precursor for starch synthesis, with its hydrolysis promoting the synthesis of amylase (**Figures 2**, **7**). Besides their roles in energy provision, these substrates, such as NAD/NADH, NADPH, FAD and carotenoid, are integral components of antioxidant systems that maintain cellular redox homeostasis by facilitating electron transfer (Li et al., 2017; Smith et al., 2021). The expression pattern of *OsNUDX23* suggests its physiological importance in energy supply and regulation of ROS levels during germination.

### OsNUDX23 regulates ROS level and starch metabolism during germination

Previous studies have demonstrated that ROS accumulate significantly during seed germination (Verma et al., 2015; Wojtyla et al., 2016). As crucial environmental sensors, ROS detect external conditions and transmit signals to cells, thereby regulating germination. The concentration of ROS is critical in controlling seed germination. It has been proposed that germination can only occur when ROS levels fall within an “oxidative window” that facilitates ROS signaling (Bailly et al., 2008), which promotes GA synthesis and ABA degradation, leading to the cell wall loosing and endosperm weakening to release seed dormancy and accelerate germination (El-Maarouf-Bouteau and Bailly, 2008; Gomes and Garcia, 2013). Levels outside the “oxidative window” might inhibit seed germination (Leymarie et al., 2012). Excessive ROS production can cause lipid peroxidation, mRNA oxidation, and protein oxidation and carbonylation, which ages the seed and inhibits the germination (Finch-Savage and Steven, 2017; Kurek et al., 2019). Our results indicate that the ROS level is higher in *Osnudx23* than WT (**Figures 5A, C, D**). Treatment with 0.1% H2O2 significantly induced coleoptile elongation at 24 h (**Figure 5B**), whereas treatment with 0.5% H_2_O_2_ inhibited this process (**Supplementary Figure S5**), highlighting the dual role of H_2_O_2_ in germination. Consistent with these conclusion, we observed that coleoptile growth was faster in *Osnudx23* than WT before 24 h imbibition (**Figures 3C-D**, **S3C-D**), but the radicle growth is inhibited in *Osnudx23* after 24 h imbibition (**Supplementary Figures S3, S4**), implying radicle are more sensitive to ROS during germination (Wojtyla et al., 2016). Plants fine-tune the balance between ROS production and scavenging for optimal seed germination (Corpas et al., 2020). OsNUDX23 plays an important role in precisely regulating ROS accumulation to maintain a balance between oxidative signaling that promotes germination and oxidative damage that inhibits or delays it.

ADPG is the direct substrate of starch synthesis, a process that occurs within chloroplasts (Stitt and Zeeman, 2012). Our results suggest that OsNUDX23 plays a role in starch synthesis and metabolism, as supported by the observations that OsNUDX23 exhibits robust hydrolase activity towards ADPG, and higher starch content was detected in *Osnudx23* seeds than WT (**Figure 2**, **7B**). Moreover, the expression levels of *OsRAmy1C* and *OsBGLU19*, both of which catalyze the degradation of starch (Karrer and Rodriguez, 1992; Muñoz-Llandes et al., 2023; Sugimoto et al., 1998), were significantly lower in *Osnudx23* mutant than WT (**Figure 7A**). In addition, the *Osnudx23* mutant exhibits significantly lower degradation rate of starch during post-imbibition compared to WT (**Supplementary Figure S7**), indicating that OsNUDX23 regulates starch metabolism in rice seeds.

### OsNUDX23 participates in plant growth and development

No significant phenotypic differences were observed between the *Osnudx23* mutants and WT at the seedling stage (**Supplementary Figure S2A**). Additionally, other agronomic traits such as effective tiller number and heading remained unchanged. However, the *Osnudx23* mutants exhibited delayed seed maturation compared to WT (**Supplementary Figure S8**), indicating that OsNUDX23 may play a role in regulating the rate of seed ripening in plants.

Plants can induce ROS accumulation under various abiotic and biotic stresses, including flooding, drought, salt stress, and pathogen attack (Babbar et al., 2020; Hipsch et al., 2021; Mittler et al., 2022). The role of OsNUDX23 in scavenging ROS suggests its potential significance in stress response. This hypothesis is supported by research demonstrating that the NUDX family plays a crucial role in stress tolerance. For example, overexpression of *AtNUDX2*, encoding ADP-ribose pyrophosphatase, enhances Arabidopsis plants’ tolerance to oxidative stress (Ogawa et al., 2009). AtNUDX6 positively regulates NPR1-dependent salicylic acid (SA) signaling pathways, significantly impacting plant immune responses (Ishikawa et al., 2010). AtNUDX8 acts as a positive regulator of defense-related pathways, contributing to plant immunity (Fonseca and Dong, 2014). Conversely, TaNUDX23 suppresses ROS accumulation to facilitate *Pst* infection (Yang et al., 2020). The potential of OsNUDX23 as a target for crop improvement is further substantiated by studies highlighting the impact of NUDIX hydrolases on plant growth and stress tolerance (Zeng et al., 2014).

In conclusion, our study elucidates the multifaceted role of OsNUDX23 in rice seed germination. By modulating ROS balance and gene expression, OsNUDX23 plays a key role in coordinating molecular events that facilitate the transition from dormancy to germination. Meanwhile, OsNUDX23 may play an important role in regulating starch synthesis and metabolism, thereby promoting rice seed ripening and in response to stresses. Further research is required to fully unravel the mechanisms by which OsNUDX23 exerts its effects and to explore its potential as a target for crop improvement.

## Data Availability

The datasets presented in this study can be found in online repositories. The names of the repository/repositories and accession number(s) can be found in the article/**Supplementary Material**.
