# Supplementary material for: OsNUDX23 regulates early seed germination by modulating ROS balance and starch metabolism in rice
Source: Front Plant Sci. 2025 Jun 13;16:1581800. doi: 10.3389/fpls.2025.1581800 (PMC12202601; doi:10.3389/fpls.2025.1581800)
Supplement: Supplementary file 1 [file SupplementaryFile1.pdf]

Supplementary Material

Supplementary Data

Additional supporting information may be found at the end of the article.

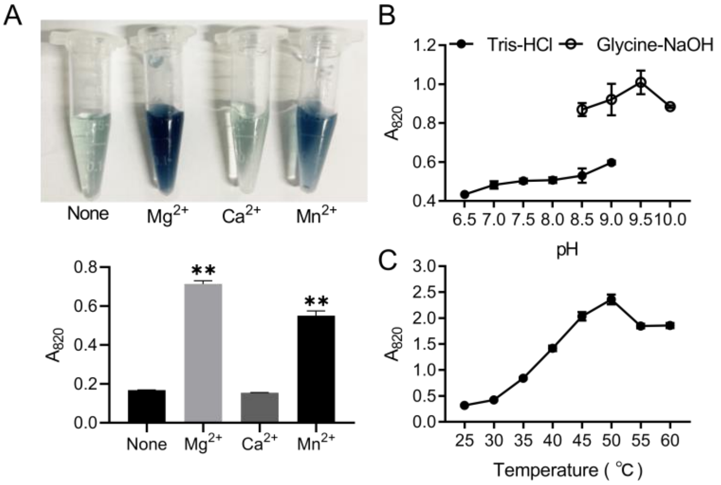

Figure S1. The optimal enzyme activity of OsNUDX23 under different ions, pH and temperature. (A) None treatment was used as control without ion. (B, C) The enzyme activity of OsNUDX23 under different pH and temperature conditions, enzyme activity was quantified by measuring absorbance at 820 nm (***p* < 0.01).

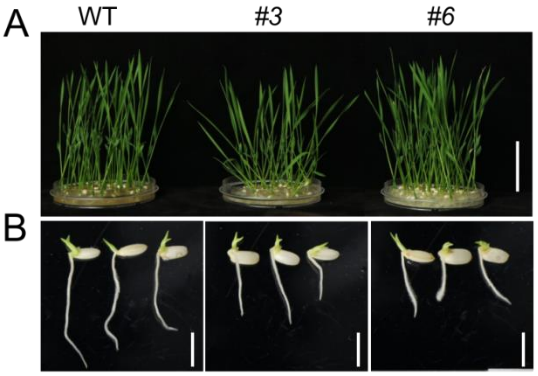

Figure S2. The phenotype of WT and *Osnudx23* lines at seedling (A) and germination (B) stage. (A) Scale bar: 5cm. (B) Scale bar: 5mm.

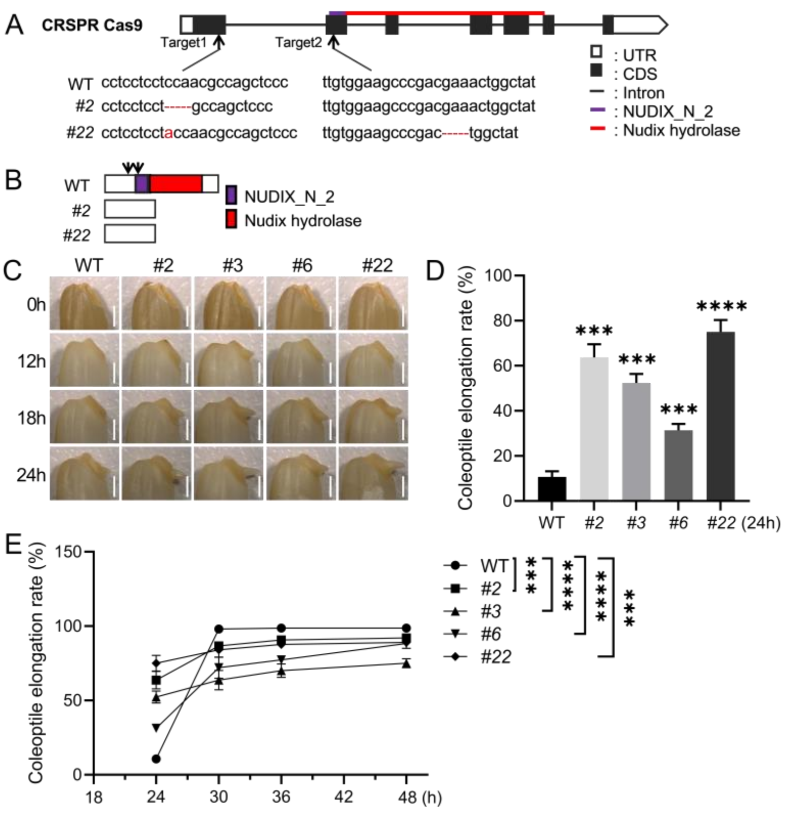

Figure S3. *OsNUDX23* affects seed coleoptile elongation in rice. (A) Schematic diagram of the *OsNUDX23* gene structure and two guides RNA target sites. (B) Schematic representation of the encoded proteins in *Osnudx23#2* and *Osnudx23#22* mutant lines compared to WT. (C) The phenotype of coleoptiles in WT and *Osnudx23* lines seeds within 24 h post-imbibition. Scale bar: 1mm. (D) Coleoptile elongation rate of WT and *Osnudx23* lines seeds at 24 h post-imbibition time. Data represent mean ± SD from three biological replicates, each with 100 seeds. Statistical significance was determined by two-sided Student's t test (***p < 0.001, ****p < 0.0001). (E) Statistics of coleoptile elongation rate of WT and *Osnudx23* lines seeds at different germination time. The length of coleoptile is ≥1mm.

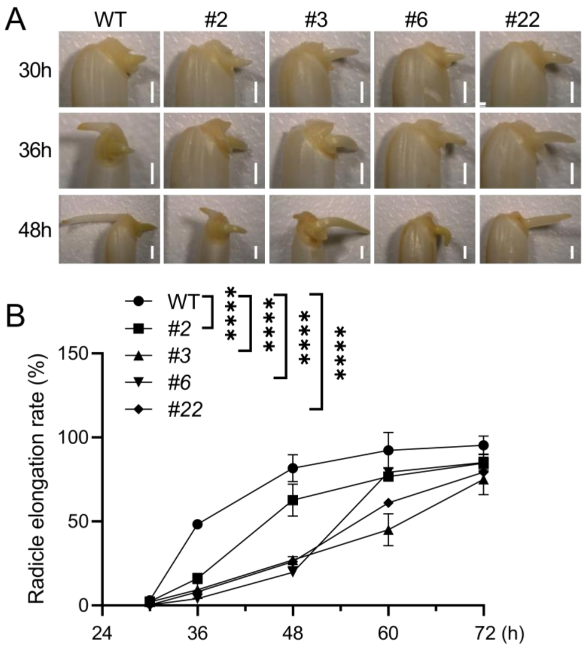

Figure S4. *OsNUDX23* affects seed germination in rice. (A) The phenotype of germination in WT and *Osnudx23* lines seeds after 24 h imbibition. Scale bar: 1mm. (B) Statistics of radicle elongation rate of WT and *Osnudx23* lines seeds after 24 h imbibition. The length of radicle is ≥1mm.

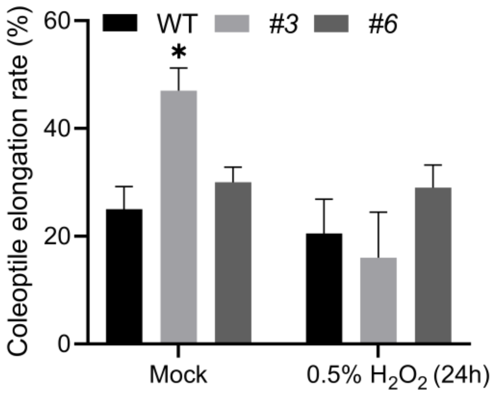

Figure S5. Coleoptile elongation was delayed by 0.5% H_2O_2. The coleoptile elongation rate of WT and *Osnudx23* lines treated with 0.5% H_2O_2. Data are presented as the mean ± SD from three biological replicates, with 100 seeds per replicate. Significance was determined by two-sided Student's t test (*$p < 0.05$).

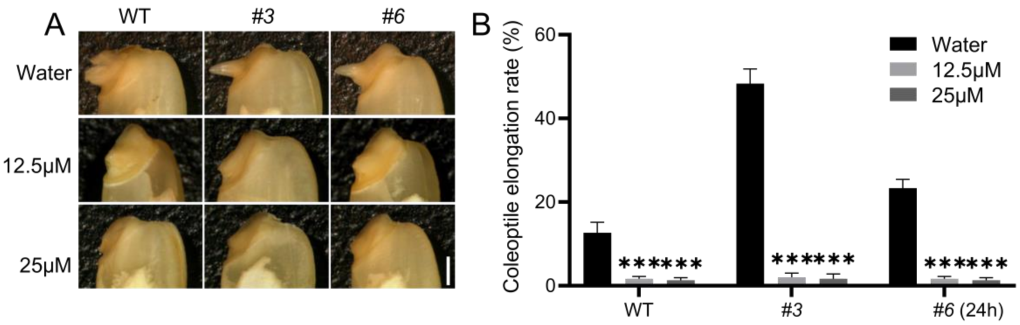

Figure S6. Coleoptile elongation was inhibited by DPI. (A) The coleoptile elongation of WT and *Osnudx23* lines incubated with 12.5 μM and 25 μM DPI. (B) Statistics of coleoptile elongation rate of WT and *Osnudx23* lines. The length of coleoptile is ≥1mm. Significance was determined by two-sided Student's t test (***$p < 0.001$).

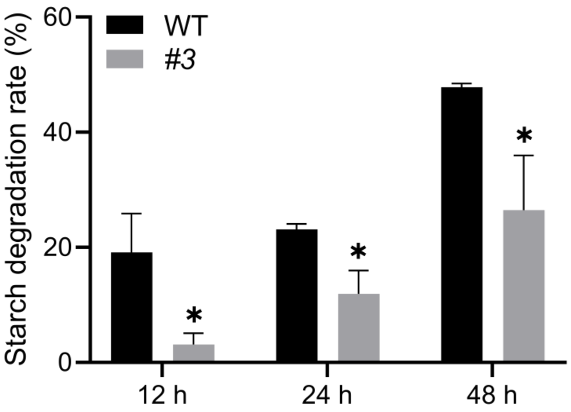

Figure S7. Starch degradation rate in WT and *Osnudx23* mutant. Starch degradation rate of seeds at 12, 24, and 48 h post-imbibition compared with 0 h in WT and *Osnudx23* mutant. Significance was determined by two-sided Student's t test (*$p < 0.05$).

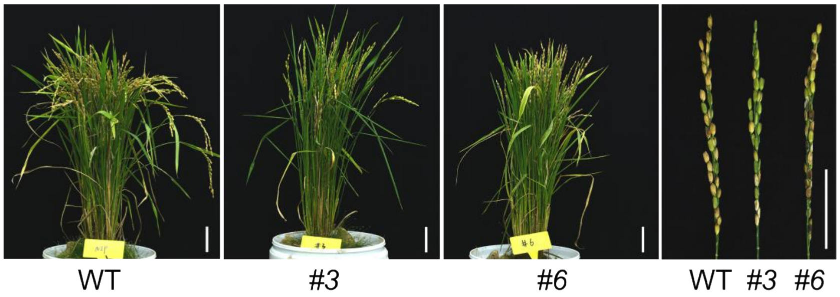

Figure S8. Phenotype of the wild-type (WT) and *Osnudx23* plants at the mature stage. Scar bar: 5 cm
